# Retraction: miR-122 Regulates Tumorigenesis in Hepatocellular Carcinoma by Targeting AKT3

**DOI:** 10.1371/journal.pone.0184778

**Published:** 2017-09-08

**Authors:** The PLOS ONE Editors

A Pfizer representative contacted the editorial office to raise concerns that there are image duplications in this article. Pfizer has undertaken a review and determined that there are duplications in Fig 4A of the article and that part of the experiments cannot be verified.

In light of the concerns identified, the authors and the PLOS ONE Editors retract this article. We did not receive any comments from Min-Jean Yin, Rounak Nassirpour or Pramod Mehta.
